# Clinical Features, Prognosis, and Long-Term Response to Ranibizumab of Macular CNVs in Pattern Dystrophies Spectrum: A Pilot Study

**DOI:** 10.1155/2021/6698522

**Published:** 2021-04-16

**Authors:** Lorenzo Casillo, Stefano Tricarico, Laura Contento, Enzo M. Vingolo

**Affiliations:** UOC Ophthalmology, Ospedale A. Fiorini Terracina, Sapienza University of Rome, Terracina 04120, Italy

## Abstract

**Introduction:**

To analyze the morphological and functional features of choroidal neovascularizations (CNVs) in eyes affected by pattern dystrophies (PD), evaluating their long-term response to intravitreal ranibizumab, and comparing them with CNVs in age-related macular degeneration (AMD). The mean goal is to identify possible disease biomarkers and to evaluate the long-term prognosis of CNVs in PD.

**Materials and Methods:**

A retrospective study of 42 patients with naïve CNV (26 PD and 16 AMD), for a total of 47 eyes (29 eyes in the PD group and 18 eyes in the AMD group). Each patient received a loading dose of ranibizumab (one monthly for three months) followed by pro re nata (PRN) reinjection protocol for a period of at least three years. Morphological OCT parameters (CRT, central retinal thickness; SRF, subretinal fluid; IRF, intraretinal fluid; SHRM, subretinal hyperreflective material; HRF, hyperreflective foci; HCD, hyperreflective crystalline deposits; cCT, central choroidal thickness; slCT, sublesional choroidal thickness; EZd, ellipsoid zone disruption; and best corrected visual acuity (BCVA in logMAR scale)) were reported at baseline and last follow-up.

**Results:**

At baseline, no significant differences were found between the two groups, except for choroidal thickness parameters that were significantly greater in the PD group (*p* = 0.009). Longitudinal PD analysis demonstrated reduction in BCVA (*p* = 0.009), decrease in CRT (*p* = 0.046), resolution of SRF in 61.6% of cases (*p* = 0.004) and SHRM in 30% (*p* = 0.034), and choroidal thinning both centrally (*p* = 0.004) and sublesional (*p* = 0.011) compared to baseline. At 3 years, the PD group received significantly more injections than the AMD (*p* = 0.011) and showed significantly thicker choroid (*p* = 0.033) and more frequent HRF (*p* = 0.006). Regarding the PD group, we found a negative correlation between age and choroidal thicknesses at baseline and at 3 years (*p* < 0.05); significant positive correlations were found between baseline BCVA and at 3 years (*p* < 0.001), BCVA at 3 years and IRF (*p* = 0.003) and SHRM at 3 years (*p* = 0.003); CRT baseline and CRT 3 years (*p* = 0.017); HCD at 3 years was associated with greater CRT (*p* = 0.04) and IRF at 3 years (*p* = 0.019).

**Conclusions:**

Early and long-term morphofunctional features of CNVs in PD and in AMD are overlapping. CNVs in PD have poorer long-term response to ranibizumab and higher choroidal thickness suggesting different pathogenetic and evolutionary mechanisms.

## 1. Introduction

Pattern dystrophies (PD) are a group of hereditary macular diseases characterized by the accumulation of lipofuscin material at the level of the retinal pigment epithelium (RPE). Based on the different RPE alteration patterns, five different subtypes of PD have been identified [1]: adult-onset foveomacular vitelliform dystrophy (AOFVD), butterfly-shaped dystrophy, reticular Sjogren dystrophy, and multifocal pattern dystrophy simulating fundus flavimaculatus and fundus pulverulentus.

The evolution of PD has been considered erroneously benign for a long time, probably due to their clinical, phenotypic, and demographic features, determining their frequent misdiagnosis with more frequent and known macular diseases, above all age-related macular degeneration (AMD). Today, we know that 42% of the affected subjects will develop severe and irreversible visual damage due to the atrophic (26%) or neovascular (18%) complications [2]. However, the etiopathogenetic bases, the morphological features, and the treatment indications and response are controversial.

This retrospective study aims to analyze the morphological and functional characteristics of choroidal neovascularizations (CNVs) in eyes affected by PD, evaluating their long-term response to intravitreal ranibizumab, and comparing them with CNVs in AMD. The mean goal is to identify possible disease biomarkers and to evaluate the long-term prognosis of CNVs in PD compared to conventional AMD.

## 2. Materials and Methods

We collected data from 42 patients with naïve CNV, of which 26 with PD and 16 with AMD, for a total of 47 eyes including 29 eyes in the PD group and 18 eyes in the conventional AMD group. The PD group included 20 subjects affected by adult-onset foveomacular vitelliform dystrophy (23 eyes), 6 with butterfly-shaped dystrophy (6 eyes), and 2 with reticular dystrophy (2 eyes). Each patient received a loading dose of ranibizumab (one monthly for three months) followed by pro re nata reinjection protocol for a period of at least three years. Patients who had previously received any treatment for CNV were excluded, as well as subjects who switched to any other anti-VEGF drug during the follow-up period. We also excluded patients affected by other macular diseases, recent anterior or posterior segment surgery, cataract surgery during the follow-up period, and positive or negative spherical refractive error >6 diopters. This study adhered to the Tenets of the Declaration of Helsinki and complied with the Health Insurance Portability and Accountability Act of 1996. This study was approved by the institutional review board (IRB) committee.

All patients underwent a complete eye exam, including best corrected visual acuity (BCVA), anterior and posterior segment examination, fundus autofluorescence (FAF), fluorescein (FA), indocyanine green angiography (ICGA), and optical coherence tomography (OCT). All data were evaluated by two independent retina experts, and in doubtful cases, a third senior expert was consulted. The diagnostic criteria for PD included the (a) absence of drusen on funduscopic examination; (b) absence of any focal RPE elevation compatible with drusen on OCT b scans and showing hyperfluorescence on FAF and FA; (c) the presence of RPE alterations of any type from focal hyperreflective thickening, RPE attenuation, to more extensive RPE alterations detectable through clinical examination, FAF, and OCT b scans; and (d) if present, the vitelliform material accrual was visible at OCT b scans as subretinal material at medium to high reflectivity and hyperfluorescent at FAF.

Morphological and functional data were reported at baseline and after a three years follow-up. BCVA was measured using the logMAR scale. Morphological parameters were collected through SD-OCT Heidelberg Spectralis (software version 5.4.7.0; Heidelberg Engineering, Germany). OCT scans were acquired with a vertical and horizontal 100 frames enhanced depth imaging (EDI) single line centered on the fovea and 20 × 15° (5.8 × 4.3 mm) rectangular scan raster centered on the fovea consisting of 25 high-resolution lines each comprising an average of 50 acquisitions. The “baseline” scan was used as a reference for all the following images acquired in the “follow-up” mode, in order to ensure reproducibility of the method. At time 0 and at 3 years, we evaluated the following parameters: central retinal thickness (CRT, automatically measured by the software), subretinal fluid (SRF, presence/absence), intraretinal fluid (IRF, including cystic formations, excluding tubulation phenomena, presence/absence), subretinal hyperreflective material (SHRM, defined as the hyperreflective material located between the neuroretina and the anterior lamina of RPE [3], presence/absence), hyperreflective crystalline deposits (HCD, diagnosed in OCT as single or multiple highly reflective lines between the RPE and Bruch's membrane [4]. On near-infrared reflectance, HCD appeared as intensively reflective plaques [5], presence/absence), hyperreflective foci (HRF, diagnosed in OCT as small and well-circumscribed hyperreflective particles in the outer retinal layers. In fundus color photography, they could appear as hard exudates; no alterations in fluorescein angiography, presence/absence), central choroidal thickness (cCT, manually measured as the distance between the outer edge of the hyperreflective line of Bruch's membrane and the inner surface of the chorioscleral junction at the level of the foveola), sublesional choroidal thickness (slCT, manually measured as the distance between the outer edge of the hyperreflective line of Bruch's membrane and the inner surface of the chorioscleral junction at the level of the center of the neovascular formation), and ellipsoid zone disruption (EZd, diagnosed in OCT as disappearance of ellipsoid zone, presence/absence). Each parameter was evaluated according to the judgment of two independent expert operators.

The data analysis was conducted using both descriptive and inferential statistics. For the descriptive statistics, quantitative and qualitative variables were used and analyzed according to a general linear model. Quantitative data were reported as mean ± standard deviation, and distribution normality was verified by the Shapiro–Wilk normality test. For the inferential statistic, significant differences between groups or intragroups were tested using the *t* test for unpaired or paired data, respectively.

The chi-square test was used to compare patient categorical variables, while the Wilcoxon test was chosen to evaluate changes in the nominal variables over time. The analysis of the relationships between variables was conducted using Spearman's tau coefficient. In all cases, *p* values less than 0.05 were considered statistically significant. Each analysis was conducted using the SPSS statistical program (ver. 25; SPSS, Inc., Chicago, IL., USA).

## 3. Results

### 3.1. Between-Groups Baseline Analysis before Treatment

Baseline demographic characteristics were assimilable between groups for age (years; PD: 74.62 ± 6.35; AMD: 75.22 ± 7.44; *p* = 0.32), sex (% female/male; PD: 61.1/38.9; AMD: 62.1/37.9; *p* = 0.95), and months of follow-up (PD: 40.72 ± 3.27; AMD: 40.11 ± 2.72; *p* = 0.55). Functional and morphological parameters of both groups were collected at the baseline (Table 1). At baseline, the visual acuity (*p* = 0.13) and the CRT (*p* = 0.13) were similar between the PD and AMD groups. Furthermore, no significant differences were found between the two groups in terms of the presence of SRF (*X*^2^ = 0.39; *p* = 0.53), IRF (*X*^2^ = 2.29; *p* = 0.13), SHRM at baseline (*X*^2^ = 0.03; *p* = 0.87), and HRF at baseline (*X*^2^ = 1.09; *p* = 0.30). At this time, HCD were not detected in any of the groups. The choroidal thickness parameters were significantly greater in the PD group than in AMD (cCT + 49.47 *µ*m and slCT + 55.12 *µ*m both with *p* = 0.009).

### 3.2. In-Group Longitudinal Long-Term Analysis before–after Treatment

At 3 years of follow-up, all the parameters considered at the baseline were reevaluated to identify any evolutions and correlations (Tables 1 and 2). In particular, the PD group demonstrated significant reduction in BCVA, considered as increasing of logMAR value (*p* = 0.009), despite a significant decrease in central retinal thickness (CRT variation *pp* = 0.046), with the resolution of SRF in 61.6% of cases (*Z* = −2.89; *p* = 0.004) and SHRM in 30% (*Z* = −2.12; *p* = 0.034). There were no significant changes in IRF (*Z* = −0.63; *p* = 0.53) and HRF (*Z* = −1.6; *p* = 0.1). The presence of HCD was detected in 3 cases, but it does not satisfy the significance criteria, despite an encouraging trend (*Z* = −1.73; *p* = 0.08). The choroid showed significant thinning both centrally (cCT V: *p* = 0.004) and sublesional (slCT V: *p* = 0.011) compared to baseline. Furthermore, no significative variation was observed in ellipsoid zone disruption (*p* = 0.21).

In the AMD group, the BCVA was substantially stable at 3-year compared to the initial value (*p* = 0.4). The CRT also showed no significant changes (*p* = 0.25), despite a significant reduction in the presence of SRF (*Z* = −2.53; *p* = 0.01) and HRF (*Z* = −2.53; *p* = 0.011). The reduction in IRF (*Z* = −0.45; *p* = 0.65) and SHRM (*Z* = −0.33; *p* = 0.74) was also not significant, as was the appearance of HCD in a single case (*Z* = −1.0; *p* = 0.32). Similar to the PD group, a significant decrease in cCT was detected (*p* = 0.012), and no significant variation of the ellipsoid zone integrity was detected (*p* = 0.08). However, unlike the PD group, the AMD subjects did not show a similar thinning of the slCT (*p* = 0.4).

### 3.3. Between-Groups Long-Term Analysis after Treatment

The final analysis of the two groups allowed us to compare the long-term response to intravitreal ranibizumab. The first interesting finding is that the PD group received significantly more injections than the AMD (*p* = 0.011). Despite this, the final BCVA between the two groups was similar (*p* = 0.95), as well as the CRT (*p* = 0.85) and the persistence of SRF (*X*^2^ = 1.18, *p* = 0.28), IRF (*X*^2^ = 0.004, *p* = 0.95), SHRM (*X*^2^ = 0.74, *p* = 0.39), and HCD (*X*^2^ = 0.33, *p* = 0.57). A highly significant difference was found in the presence of HRF, more frequent in the PD group (*X*^2^ = 7.50; *p* = 0.006). Finally, the cCT of the PD group was significantly thicker than the AMD group (*p* = 0.033); therefore, the cCT variance did not differ between groups (*p* = 0.21).

### 3.4. PD In-Group Quantitative and Qualitative Correlations

Each quantitative and qualitative parameter was cross-compared to each other, but only the most clinically relevant correlations were reported. Analyzing the PD group, we found a negative correlation between age and cCT at baseline and cCT at 3 years (respectively, *R* = −0.46, *p* = 0.012; *R* = −0.58, *p* = 0.001) and also showed in the sublesional measurements slCT baseline and slCT at 3 years (respectively, *R* = −0.41, *p* = 0.027; *R* = −0.51, *p* = 0.004). Moreover, we found a significant positive correlation between BCVA at baseline and at 3 years (*R* = +0.64; *p* < 0.001) and between BCVA at 3 years (considered as logMAR value) and the presence of IRF (*R* = + 0.53, *p* = 0.003) and SHRM at 3 years (*R* = +0.54, *p* = 0.003). Interestingly, we noticed no correlation between BCVA 3 years and SRF 3 years (*R* = −0.28, *p* = 0.14), whereas the CRT baseline and CRT 3 years showed a positive correlation, with *R* = +0.44 and *p* = 0.017. However, except for the correlations between CRT and SHRM at baseline (*R* = +0.54, *p* = 0.003) and SRF 3 years-cCT 3 years (*R* = +0.44, *p* = 0.016), none of the morphological parameters considered showed a predominant role in influencing the CRT in the PD subgroup. The presence of SHRM at baseline correlated not only with the CRT baseline but also with the presence of IRF at 3 years (*R* = +0.37, *p* = 0.048) and SHRM 3 years (*R* = +0.50, *p* = 0.005). Of note, the presence of HCD at 3 years was associated with greater CRT (*R* = +0.38, *p* = 0.04) and IRF at 3 years (*R* = +0.44, *p* = 0.019).

On the AMD group, we observed positive correlations between choroidal thickness parameters longitudinally, cCT baseline-cCT 3 years (*R* = +0.83, *p* < 0.001), slCT baseline-slCT 3 years (*R* = +0.91, *p* < 0.001) but also cCT baseline-slCT baseline (*R* = +0.80, *p* < 0.001), cCT 3 years-slCT 3 years (*R* = +0.86, *p* < 0.001). Contrariwise to the PD group, no correlations were found between choroidal thickness and age: cCT at baseline-age (*R* = −0.33, *p* = 0.18) and cCT 3 years age (*R* = −0.27; *p* = 0.27). The final BCVA (logMAR at 3 years) was associated with the presence of SHRM at 3 years (*R* = +0.62, *p* = 0.007), as seen in the PD subgroup. The CRT baseline increased with the presence of IRF baseline (*R* = +0.49, *p* = 0.039), while after 3 years, it was influenced by the presence of both SRF (CRT 3 years-SRF 3 years: *R* = +0.63, *p* = 0.005) and HRF (*R* = +0.56, *p* = 0.016). In this regard, the increasing SRF at 3 years was strongly accompanied by the presence of HRF (*R* = +0.66, *p* = 0.003).

## 4. Discussion

The clinical and morphological features of the two groups were similar at baseline exhibiting no differences in visual acuity, central macular thickness, subretinal or intraretinal fluid, subretinal material, and hyperreflective crystalline deposits, with the only exception of choroidal thicknesses. The PD subgroup showed thicker choroidal thicknesses both centrally and underneath the neovascular lesion at baseline ad at last follow-up. These findings appear to be comparable with similar but not analogous findings already present in the literature.

In fact, while Palacios et al. in 2016 demonstrated a comparable choroidal thickness between PD patients and healthy population [6], Coscas et al. highlighted how subjects with wet AMD had thinner choroids compared to adult-onset foveomacular vitelliform dystrophy (AOFVD) not yet complicated by CNV, indicating a possible role in the differential diagnosis [7]. Grenga et al. confirmed these findings and observed a progressive increase in the choroidal thickness based on the evolutive stage of the not yet neovascular disease, assuming a possible prognostic and pathogenetic role of the choroid in the AOFVD [8]. In this regard, our data show a moderate agreement between choroidal thickness and subretinal fluid at last follow-up. This correlation was not observed in the AMD group; therefore, this finding may suggest the possible role of choroidal hyperpermeability in sustaining the subretinal fluid often present even without detectable neovascularization in AOFVD [9–12].

It is reasonable to hypothesize that the variation in choroidal thickness based on the evolutionary stage may represent a compensatory mechanism for the elimination of vitelliform/lipofuscin material by the hypertrophic pigment epithelium. Therefore, it would be plausible that the thickening of the outer retinal vessels in the Haller layer could cause secondary damage to the overlying choriocapillaris network, triggering ischemic phenomena responsible for subsequent neovascularization. This may represent an alternative pathogenic mechanism in contraposition to the AMD, where the progressive choroidal thinning is mainly related to aging [13]. Our data confirm the progressive choroidal thinning over time in the AMD group, as previously reported in the literature [14, 15]. In AMD eyes, the choroid and the choriocapillaris present progressive thinning inducing ischemic alterations of the microcirculation, similar to other vascular systemic diseases (e.g., atherosclerosis and cerebrovascular insufficiency). In the PD eyes, despite a progressive choroidal thinning similar to the AMD group, likely due to aging and/or bevacizumab treatment, the choroid remained thicker through the 3 years of follow-up. This finding further corroborates the different role of the choroid in driving the development of macular complications in such cases.

The baseline visual acuity was similar in both groups; however, while the PD group had very homogeneous values among the enrolled subjects (logMAR baseline: mean = 0.55; SD = 0.15), the AMD group showed markedly greater variability (mean = 0.72; SD = 0.44). The visual acuity after 3 years showed clearly more similar values compared to the initial data: logMARPD (mean = 0.86; SD = 0.66); logMARAMD (mean = 0.85; SD = 0.58). In fact, the longitudinal evaluation of the PD group demonstrated a significant loss of vision over the 3 years, while the functional progression in the AMD group was not significant.

Based on the arguments, our data suggest that CNVs in AMD and PD may have different evolution trends, as already partially demonstrated in the literature [16]. Another interesting finding is that both groups, AMD and PD, showed worse visual acuity at 3 years when subretinal hyperreflective material and, but only in the PD group, intraretinal fluid were present. A comparison with other intravitreal medications would be required, in order to evaluate differences in terms of functional and morphological responses.

With regard to the therapeutic response of the PD group, it is also interesting to observe that the average lowering of visual acuity is unexpectedly associated to a significant improvement in morphological parameters, such as central macular thickness, presence of fluid and subretinal hyperreflective material, and ellipsoid zone integrity but with persistence of hyperreflective intraretinal foci. The AMD group, on the other hand, shows greater volubility in the variation of the central thickness, despite the resolution of subretinal fluid in more than 50% of the subjects, associated to a reduction of hyperreflective foci. In this regard, it is interesting to observe that in the AMD group, the permanence of subretinal fluid and hyperreflective foci are associated with each other and with a higher central retinal thickness.

It is important to underline that a good short-term morphofunctional response of CNVs in PD is commonly reported in literature [17–19]. Many long-term follow-up studies, on the other hand, confirm a morphological improvement, nevertheless emerging divergences in terms of functional recovery [16, 20]. This apparent difference between functional and morphological features could depend on several factors, for example, the trend of these lesions to early damage the photoreceptors that were already suffering due to the presence of accumulation of vitelliform material and/or dysfunction of the EPR; an alternative explanation is the greater tendency to fibrosis than conventional CNVs. This point would deserve further investigation with other observational studies with adequate follow-up time.

Another noteworthy aspect is the presence of hyperreflective crystalline deposits. These cholesterol-based crystalline formations [5] were not observed in any patient at baseline, but appeared only in the long term, in double (albeit not significant) percentages in the PD group compared to AMD. Furthermore, in the PD group, HCD was associated with the permanence of intraretinal fluid and the final central macular thickness, confirming and integrating their already known nature of the negative prognostic factor, demonstrated to date in relation to the dry forms of AMD [4, 21]. Furthermore, their presence could represent a further point in support of the greater tendency to atrophic evolution of the disease; this evidence was also observed in the course of avascular lesions, such as drusenoid detachments of the retinal pigment epithelium. This could open a new scenario for the treatment of CNV associated with PD, which should not be “overtreated” to avoid a further atrophic effect. In fact, we reported a higher number of injections at 3 years in the PD group. One of the possible reasons for overtreatment lies in the hypothesis that a part of subretinal fluid could be due to RPE pump failure instead of CNV activity.

Data collected allow us to state that both at baseline and at 3 years follow-up, morphological and functional parameters between the two groups are similar, with the exception of a greater central choroidal thickness and a greater percentage of hyperreflective foci in the PD group; however, the real difference between the two groups lies in the type of progression and response to therapy.

The limit of our study is the retrospective nature and the small sample size, negatively influenced by the stringent inclusion criteria regarding the use of monotherapy and the availability of complete multimodal imaging. Furthermore, the choice of ranibizumab as the only drug and the administration with the PRN protocol are limiting choices, but necessary to guarantee homogeneity of the data collected, which come, in part, from databases created when more modern drugs and protocols, such as aflibercept and “Treat and extend,” had not yet entered common clinical practice.

## 5. Conclusions

The differential diagnosis between neovascular age-related macular degeneration and CNV on pattern dystrophies is still challenging even for the most experienced retinologists. Our study highlights the clinical and morphological overlap of the two forms both in the onset and in the long-term, confirming the necessity of complete multimodal imaging in order not to incur erroneous diagnoses. Furthermore, we report a poor therapeutic response at 3 years of the forms associated with pattern dystrophies compared to those in AMD which, instead, show a tendency to stabilization. Finally, the higher choroidal thickness could open new perspectives in understanding the pathogenetic and evolutionary mechanisms underlying the development of neovascularizations in the eyes affected by pattern dystrophies.

The main limitation of our study is represented by low and poorly balanced numerosity of the two groups. This is due to our strict inclusion criteria, including age, complete multimodal imaging, PRN monotherapy administration, and no surgery in the follow-up period.

Further studies are needed to deepen these topics.

## Figures and Tables

**Table 1:** PD and AMD parameters at baseline and after 3 years.

	Baseline			3 years		
	PD	AMD	*p* value	PD	AMD	*p* value
LogMAR, mean ± SD	0.55 ± 0.15	0.72 ± 0.44	0.13	0.86 ± 0.66	0.85 ± 0.58	0.95
CRT, *µ*m, mean ± SD	413.66 ± 92.54	431.56 ± 142.39	0.13	365.90 ± 135.28	374.61 ± 162.27	0.85
SRF (%)	89.66	83.33	0.53	55.2	38.9	0.28
IRF (%)	31.03	44.44	0.35	37.9	38.9	0.95
SHRM (%)	69.0	66.7	0.87	48.3	61.1	0.39
HRF (%)	96.55	88.89	0.3	82.8	44.4	0.006
HCD (%)	0	0	-	10.3	5.6	0.57
cCT, *µ*m, mean ± SD	214.14 ± 79.13	164.67 ± 45.81	0.009	186.66 ± 78.20	146.06 ± 48.33	0.033
slCT, *µ*m, mean ± SD	211.44 ± 85.27	156.33 ± 53.07	0.009	183.52 ± 89.18	151.33 ± 58.08	0.14
EZd (%)	55.17	66.7	0.43	68.96	83.33	0.27
INJ N, mean ± SD	—	—	—	8.72 ± 1.16	7.28 ± 2.54	0.011

PD, pattern dystrophies; AMD, age-related macular degeneration; LogMAR, visual acuity; CRT, central retinal thickness; SRF, subretinal fluid; IRF, intraretinal fluid; SHRM, subretinal hyperreflective material; HRF, hyperreflective foci; HCD, hyperreflective crystalline deposits; cCT, central choroidal thickness; slCT, sublesional choroidal thickness; EZd, ellipsoid zone disruption; SD, standard deviation; *µ*m, micrometer. The chi-square test is for quantitative data, and the unpaired-*t* test is for qualitative data.

**Table 2:** Longitudinal analysis.

	PD		AMD	
	Variation	*p* value	Variation	*p* value
LogMAR, mean ± SD	0.31 ± 0.6	0.009	0.13 ± 0.65	0.4
CRT, *µ*m, mean ± SD	−47.75 ± 123.07	0.046	−56.94 ± 204.28	0.25
SRF (%)	−61.6	0.004	−53.3	0.011
IRF (%)	22.1	0.52	−22.1	0.65
SHRM (%)	−30	0.034	−12.5	0.74
HRF (%)	−14.3	0.1	−50.0	0.011
HCD (%)	10.3	0.08	5.6	0.32
cCT, *µ*m, mean ± SD	−27.48 ± 47.26	0.004	−18.61 ± 27.88	0.012
slCT, *µ*m, mean ± SD	−27.93 ± 55.27	0.011	−5.0 ± 24.39	0.4
EZd (%)	13.79	0.21	16.63	0.08

PD, pattern dystrophies; AMD, age-related macular degeneration; LogMAR, visual acuity; CRT, central retinal thickness; SRF, subretinal fluid; IRF, intraretinal fluid; SHRM, subretinal hyperreflective material; HRF, hyperreflective foci; HCD, hyperreflective crystalline deposits; cCT, central choroidal thickness; slCT, sublesional choroidal thickness; EZd, ellipsoid zone disruption; SD, standard deviation; *µ*m, micrometer. The chi-square test is for quantitative data, and the unpaired-*t* test is for qualitative data.

## Data Availability

The data used to support the findings of this study are available from the corresponding author upon request.
